# Therapeutic Efficacy and Safety of Paeoniae Radix Rubra Formulae in Relieving Hyperbilirubinemia Induced by Viral Hepatitis: A Meta-Analysis

**DOI:** 10.3389/fphar.2016.00063

**Published:** 2016-03-22

**Authors:** Yin-Qiu Huang, Xiao Ma, Jian Wang, Yan-Ling Zhao, Jia-Bo Wang, Zhe Chen, Yun Zhu, Li-Mei Shan, Shi-Zhang Wei, Ji Wang, Xiao-He Xiao

**Affiliations:** ^1^Pharmacy College, Chengdu University of Traditional Chinese MedicineChengdu, China; ^2^Department of Pharmacy, 302 Military Hospital of ChinaBeijing, China; ^3^China Military Institute of Chinese Medicine, 302 Hospital of People's Liberation ArmyBeijing, China; ^4^Department of Integrative Medical Center, 302 Hospital of People's Liberation ArmyBeijing, China; ^5^Department of Evidence-Based Medicine and Clinical Epidemiology, West China Hospital, Sichuan UniversityChengdu, China

**Keywords:** Paeoniae Radix Rubra formulae, efficacy and safety, meta-analysis, hyperbilirubinemia, viral hepatitis, high dose Paeoniae Radix Rubra

## Abstract

**Objective:** Hyperbilirubinemia is one of the most devastating pathologies induced by various liver diseases. Formulae related to *Paeoniae Radix Rubra* (PRR) at high doses have been applied to treat hyperbilirubinemia in traditional Chinese medicine (TCM). The aim of this systematic review and meta-analysis is to assess the efficacy and safety of formulae relevant to high-dose PRR in patients suffering from hyperbilirubinemia induced by viral hepatitis.

**Methods:** We performed a meta-analysis of randomized-controlled clinical trials to evaluate the efficacy and safety of formulae that apply a high dose of PRR for hyperbilirubinemia. Seven databases were searched until April, 2015. All studies were included according to detailed criteria and assessed for methodological quality. The outcome measurements were recorded for further analysis using the RevMan 5.2.11 software.

**Results:** Fifteen articles involving 1323 patients with hyperbilirubinemia were included. Formulae with high-dose PRR might promote the efficacy of either a combined application ([OR: 3.98, 95% CI (2.91, 5.43)]; *P* < 0.01) or a single application ([OR: 4.00, 95% CI (1.50, 10.68)]; *P* < 0.01) for hyperbilirubinemia. The indices of TBIL, ALT, and AST significantly decreased ([MD: –75.57, 95% CI (−94.88, −56.26)], [MD: −26.54, 95% CI (−36.19, −16.88)], and ([MD: −28.94, 95% CI (−46.26, −11.61)]; *P* < 0.01), respectively. In addition, formulae with high-dose PRR could enhance the treatment efficacy of hyperbilirubinemia triggered by hepatitis B ([OR: 2.98, 95% CI (1.75, 5.05)]; *P* < 0.01). Furthermore, the efficacy was enhanced with an increasing dosage of PRR. Two articles reported that no side effects occurred in clinical trials, and three studies noted that patients presented light digestive tract symptoms.

**Conclusion:** Formulae relevant to high-dose PRR ameliorate hyperbilirubinemia and might constitute a promising therapeutic approach. For widespread acceptance by practitioners, more rigorously designed multicenter, double-blind, randomized, and large-scale controlled trials are required.

## Introduction

Hyperbilirubinemia presenting high serum bilirubin or severe jaundice is one of the most devastating pathologies in patients with various liver diseases (Duan et al., 2006). Continuous hyperbilirubinemia without proper treatment commonly leads to hepatocyte edema, necrosis, inflammation, and even liver cirrhosis, which can directly threaten quality of life (Garg et al., 2011). Current therapies for hyperbilirubinemia mainly focus on a combination of decreasing serum bilirubin, liver protection, regulating transaminase levels, and nutrition support. However, there are unstable curative rates as well as side effects in the clinic. Ursodeoxycholic acid (UDCA) and Ademetionine are the first-line drugs used to decrease serum bilirubin. The inconsistent efficiency and expensive medical costs are common obstacles for some patients (Hou et al., 2010). Potassium magnesium aspartate as an adjuvant is limited by its efficacy in decreasing serum bilirubin. In addition, glucocorticosteroid therapy, due to side effects and the possibility of symptom rebound, is controversial, particularly when treating hyperbilirubinemia for a long duration (Zhang and Shen, 2011).

**Figure 1:**
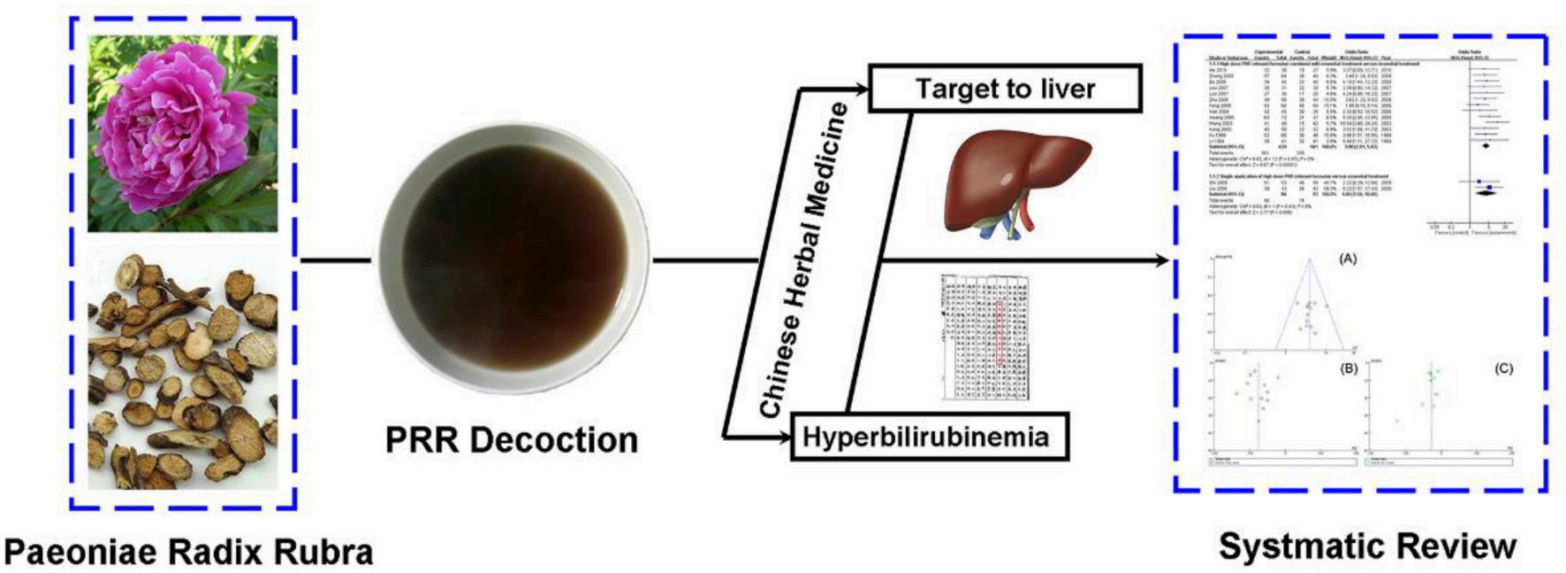
**Study flow of Paeoniae Radix Rubra in hyperbilirubinemia**. It demonstrated that PRR has been used for the treatment of blood stasis for hundreds of years, as evidenced by records from Ben Cao Gang Mu in 1578 (Ming dynasty). This study was performed to assess the clinical value of high-dose PRR-relevant formulae in the treatment of hyperbilirubinemia.

Growing evidence indicates that traditional Chinese medicine (TCM), as a complementary and alternative therapy, has an important role in hyperbilirubinemia or jaundice treatment (Li et al., 2008). The decoctions of Yin Chen Hao, Yin Chen Si Ni, Da Huang Xiao Shi, Zhi Zi Bai Pi, and Chi Dan Tui Huang have been proven to effectively decrease serum bilirubin and protect hepatocytes (He et al., 2003; Zhou, 2010). In particular, several formulae that reverse blood stasis are commonly applied in hyperbilirubinemia. *Paeoniae Radix Rubra* (PRR, Chishao in Chinese), the dried root of *Paeonia lactiflora* Pall, has been used for thousands of years in TCM to treat blood stasis. A series of PRR-relevant formulae, due to their significant function as blood invigorators, have been used as treatments for jaundice for decades and have obtained satisfactory efficacy (Ding et al., 2003). Currently, these formulae have been reported as promising therapies in the clinic and are widely used in China. However, a systematic review on formulae relevant to high-dose PRR for treating hyperbilirubinemia has not been reported. Therefore, this meta-analysis of randomized controlled trials (RCTs) was conducted to assess the clinical value of formulae relevant to high-dose PRR in treating hyperbilirubinemia and to provide a possible complementary and alternative therapy for widespread use by practitioners (Figure 1).

## Methods

### Search strategy

Comprehensive searches were performed in seven databases by two researchers. The databases included PubMed, Embase, Cochrane Library, Chinese Biomedical Database (CBM), Wanfang, VIP medicine information system (VMIS), and China National Knowledge Infrastructure (CNKI) from inception to April, 2015. Search terms included “TCM” or “PRR” or “Chishao” “*Paeonia lactiflora* pall” or “*Paeonia veitchii* Lynch”, and “severe jaundice,” or “hyperbilirubinemia,” and “viral hepatitis” according to databases in the Chinese and English languages. The first author, year of publication, title, and journal name from searched articles were recorded.

### Exclusion criteria

Studies with the following criteria were excluded: (1) reviews, non-clinical studies, and case observations, (2) the absence of Randomized controlled trials (RCTs), or (3) interventions with another TCM formulae (not including extracted agents) or acupuncture therapies in the control group.

### Inclusion criteria

According to the advice of a liver specialist, the inclusion criteria were designed as follows: (1) RCTs with patients diagnosed as having hyperbilirubinemia by meeting the criteria of Viral Hepatitis Prevention and Treatment Programs (VHPTP) version 2010, 2005, 2000, 1995, or 1990. (2) Studies must have claimed that patients developed hyperbilirubinemia due to viral hepatitis. In addition, participant serum TBIL was above 171 μmol/L before treatment. (3) PRR served as the main component in the formulae and the high dosage was defined as >25 g (two times higher than the prescribed maximum dose of Chinese Pharmacopeia 2010 version). (4) High-dose PRR-relevant formulae alone or as a combined application served as the trial group. Placebo or conventional therapy served as the control group. (5) Outcome measurements must have included one or more of the following indices: total effective rate, alanine aminotransferase (ALT), aspartate aminotransferase (AST), total bilirubin (TBIL), direct bilirubin (DBIL), γ-glutamyl transferase (γ-GT), albumin (ALB), and prothrombin time (PT). (6) The total efficacy was coincident with the Guidance for Clinical Research on New Drugs of TCM, i.e., recovery as evidenced by the absence of symptoms and TBIL decreasing to nearly normal levels. Effectiveness was identified by ameliorated symptoms and TBIL decreasing below half of the original value (85.5 μmol/L). Invalidation was defined as symptoms and TBIL remaining unchanged or worsening.

### Data extraction and risk of bias assessment

Data extraction and quality assessment were independently performed by two researchers, and disagreements were resolved by consensus. Detailed data such as study design, participants' information, interventions, outcome measures, and adverse events were extracted to a conclusive table.

The Cochrane risk of bias tool was used to access the methodological quality of the included RCTs. There were six domains, including random sequence generation (selection bias), allocation concealment (selection bias), blinding of participants and personnel (performance bias), blinding of outcome data (attrition bias), incomplete outcome data (attrition bias), and selective reporting (reporting bias).

### Data analysis

Statistical analysis was performed using Cochrane RevMan 5.2.11 (Cochrane Collaboration). Dichotomous data were presented as the odds ratio (OR) and continuous variables as the mean difference (MD) with 95% confidence intervals (95% CI). Statistical heterogeneity was assessed by Cochrane's Q-test. Data with low heterogeneity (*P* ≥ 0.10 and *I*^2^ ≤ 50%) were analyzed by a fixed-effects model, whereas a random-effect model was used for data with high heterogeneity. A funnel plot was used to assess potential publication bias.

## Results

### Characteristics of the included studies

**Figure 2:**
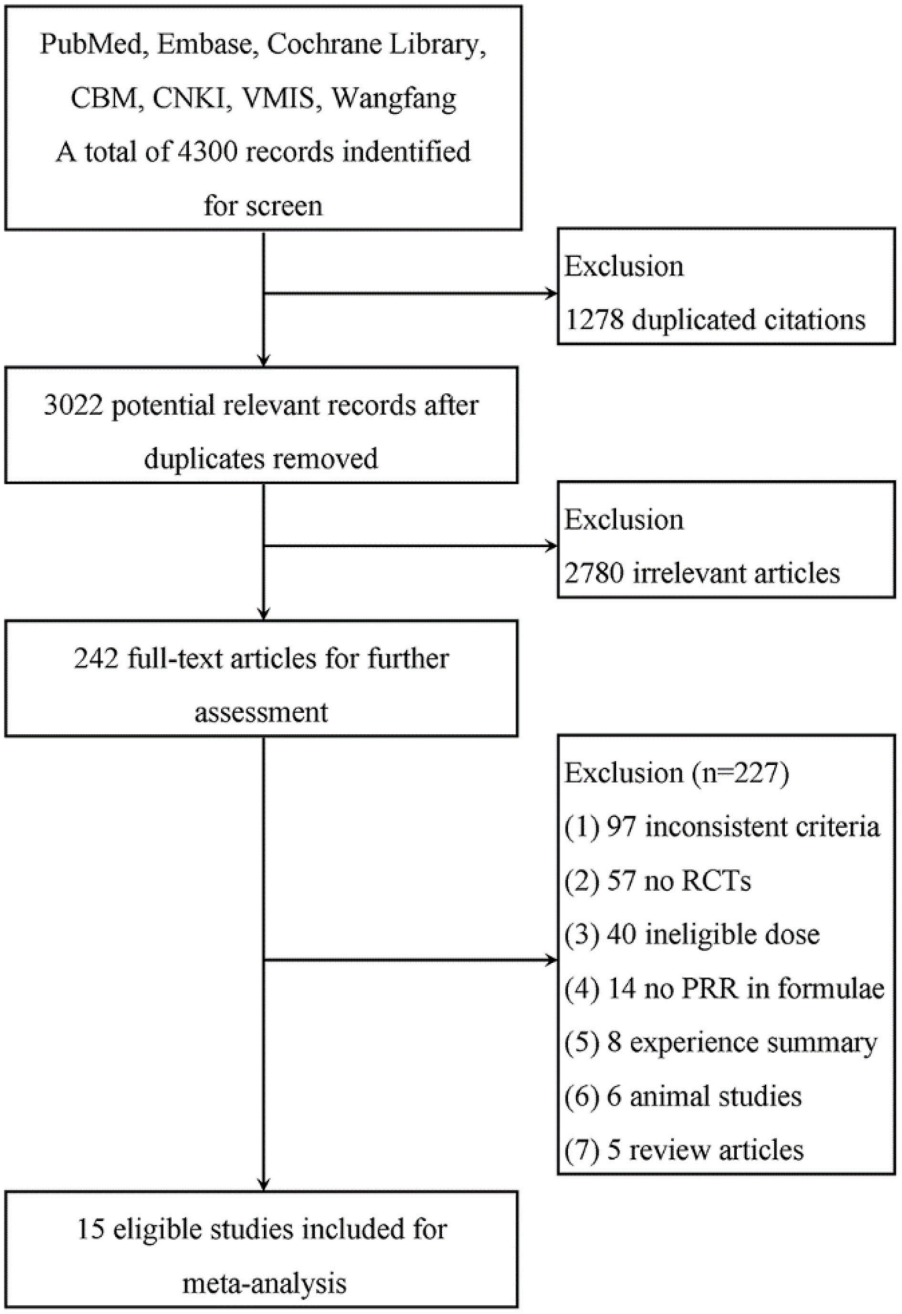
**Flow diagram of the study selection process for the systematic review**. As shown, our initial searches yielded 4300 records. The full texts of 242 articles were retrieved for detailed assessment after exclusion. Of these, 227 studies were subsequently excluded because they did not meet the inclusion criteria; 15 eligible studies were identified.

A total of 4300 publications from 7 databases were identified for the initial screen. We removed 1278 duplicated citations, and 2780 were excluded due to irrelevance. The full texts of 242 articles were retrieved for detailed assessment. Of the remaining articles, there were 57 non-randomized controlled studies, 6 animal studies, 97 inconsistent criteria, 40 ineligible PRR doses in the formulae or no mention of dosage, 14 with no PRR in the formulae, 8 summaries, and 5 review articles. Fifteen studies were ultimately used for further meta-analysis (Figure 2).

Fifteen articles involving 1323 subjects with hyperbilirubinemia (729 cases in the trial group, 594 cases in the control group) were included in this study. Because most of the formulae-form therapy was applied in TCM, the 15 studies that had been screened were all published in Chinese. There was no significance in terms of age and sex between these two groups (Table 1). Formulae with high-dose PRR with essential treatment or as a single application were used in the trial group, whereas essential treatment was applied in the control group in all studies. The dose of PRR in the formulae ranged from 30 to 200 g. The treatment duration described in the articles ranged from 2 weeks to 60 days, and 5 articles (Li and Gu, 1994; Xu et al., 1999; Wang et al., 2003; Lou et al., 2007; Bo, 2008) reported adverse events and side effects. No trial reported a follow-up (Table 2).

**Table 1:** **Characteristics of included studies**.

**Author and published year(reference)**	**Cases T/C**	**Diagnostic standard**	**Age(years) Range, mean**	**Sex Male/female**
He, 2010	36/27	VHPTP(2005)+TBIL	T:9–62, 36	T:27/9
			C:13–65, 42	C:16/11
Shi et al., 2009	53/50	VHPTP(2000)+TBIL	T:45.3	T:28/25
			C:46.9	C:27/23
Zhang, 2009	64/40	VHPTP(2000)+TBIL	T:18–56, 41	T:40/24
			C:19–58, 43	C:28/12
Bo, 2008	40/40	VHPTP(2000)+TBIL	T:20–63, 44.4	T:30/10
			C:18–62, 46.3	C:28/12
Luo, 2007	31/30	VHPTP(2000)+TBIL	T:21–50, 35.6	T:20/11
			C:22–52, 36.7	C:20/10
Luo, 2007	30/25	VHPTP(2000)+TBIL	T: 26–62, 36.3	T:23/7
			C:25–60, 34.2	C:21/4
Feng et al., 2006	60/60	VHPTP(2000)+TBIL	T:38.6	T:45/15
			C:37.8	C:47/13
Han et al., 2006	45/35	VHPTP(2000)+TBIL	T/C:41.9	65/15
Zhu et al., 2006	56/44	VHPTP(2000)+TBIL	T:33.5	T:45/11
			C:34.2	C:38/6
Huang and Yang, 2005	72/37	VHPTP(2000)+TBIL	T:19–58, 40.5	T:46/26
			C:18–56, 38.6	C:23/14
Kong et al., 2003	50/32	VHPTP(1995)+TBIL	T:12–74, 45.7	T:44/6
			C:14–72, 46.9	C:28/4
Wang et al., 2003	48/42	VHPTP(2000)+TBIL	37	NR
Liu, 2000	43/43	VHPTP(1990)+TBIL	T/C:12–58, 36	60/26
Xu et al., 1999	60/48	VHPTP(1995)+TBIL	T:22/58, 41	T:44/16
			C:21–56, 38	C:32/16
Li and Gu, 1994	41/41	VHPTP(1990)+TBIL	T/C:18–50	T:28/13
				C:27/14

*T, trial group; C, control group. NR, no report*.

**Table 2:** **Intervention and outcome measures of included studies**.

**Study ID**	**Intervention**		**Duration/follow up**	**Adverse events**	**Outcome measures**
	**Trials group (high-dose PRR-relevant formulae)**	**Control group (essential treatment)**			
He, 2010	Tui Huang Decoction (PRR 60 g)+Essential treatment	Diammonium glycyrrhizinate +Vitamin+Inosine+Ku Huang injection potassium magnesium aspartate+Reduced glutatione	6 weeks/NR	NR	Total efficacy rate, ALT, TBIL
Shi et al., 2009	Qing Re Liang Xue Jie Du Decoction (PRR 30 g)	Hepatocyte growth promotion +Liver protection+Improving minicirculation	2 weeks/NR	NR	Total efficacy rate, ALT, AST, TBIL, γ-GT, PT
Zhang, 2009	Tui Huang Decoction (PRR 30 g)+Essential treatment	Diammonium glycyrrhizinate +Inosine+Vitamin+Reduced glutatione+glucuronolactone	30 days/NR	NR	Total efficacy rate, ALT, AST, TBIL, DBIL
Bo, 2008	Jia Wei Wen Dan Decoction (PRR 30 g)+Essential treatment	Diammonium glycyrrhizinate +Potassium magnesium aspartate+Vitamin	30 days/NR	NO	Total efficacy rate, ALT, ALB, TBIL
Luo, 2007	Tui Huang Decoction (PRR 30 g)+Essential treatment	Diammonium glycyrrhizinate +Potassium magnesium aspartate+Vitamin	6 weeks/NR	T: 1 case diarrhea C: NO	Total efficacy rate, ALT, AST, TBIL, DBIL
Luo, 2007	Jie Du Huo Xue Decoction (PRR 60 g)+Essential treatment	Diammonium glycyrrhizinate +Potassium magnesium aspartate +Reduced glutatione	30 days/NR	NR	Total efficacy rate, ALT, TBIL, ALB
Feng et al., 2006	Da Huang Chi Shao Decoction (PRR 30-60 g)+Essential treatment	Liver Protection+symptom Treatment	60 days/NR	NR	Total efficacy rate, ALT, AST, TBIL, γ-GT
Han et al., 2006	Chi Ze Decoction (PRR 100 g) +Essential treatment	Liver Protection+symptom Treatment	1 month/NR	NR	Total efficacy rate, ALT, TBIL
Zhu et al., 2006	PRR 200 g+Essential treatment	Dan Shen injection+Vitamin	1 month/NR	NR	Total efficacy rate
Huang and Yang, 2005	Liang Xue Hua Yu Decoction(PRR 50 g)+Essential treatment	Vitamin+Hepatocyte growth-promoting factors+Potassium magnesium aspartate+ALB	30 days/NR	NR	Total efficacy rate, ALT, AST, TBIL, DBIL
Kong et al., 2003	PRR Relevant Formulae(PRR 30-60 g)+Essential treatment	Diammonium glycyrrhizinate +Potassium magnesium aspartate+Vitamin+Hepatocyte growth-promoting factors	6 weeks/NR	NR	Total efficacy rate, ALT, TBIL, ALB, PT
Wang et al., 2003	Tui Gao Huang Decoction(PRR 120 g)+Essential treatment	Vitamin+Adenosine disodiu+Yin Zhi Huang injection	4 weeks/NR	T: Few with light digestive tract side effect C: NO	Total efficacy rate, ALT, TBIL, ALB, PT
Liu, 2000	PRR relevant formulae(PRR 100–150 g)	Potassium magnesium aspartate +Vitamin+Bifendate	2 weeks/NR	NR	Total efficacy rate
Xu et al., 1999	Chi Zhi Huang Decoction(PRR 90 g)+Essential treatment	Potassium magnesium aspartate +Vitamin+Adenosine Disodiu+Glucuronolactone+ Silymarin	30 days/NR	T: Few with light digestive tract side effect C: NO	Total efficacy rate
Li and Gu, 1994	PRR relevant formulae(PRR 60–120 g)+Essential treatment	Silymarin+Vitamin+Inosine+ Potassium magnesium aspartate+Yin Zhi Huang injection	30 days/NR	NO	Total efficacy rate, TBIL

T, trial group, C, control group. NR, no report.

### Methodological quality of included trials

**Figure 3:**
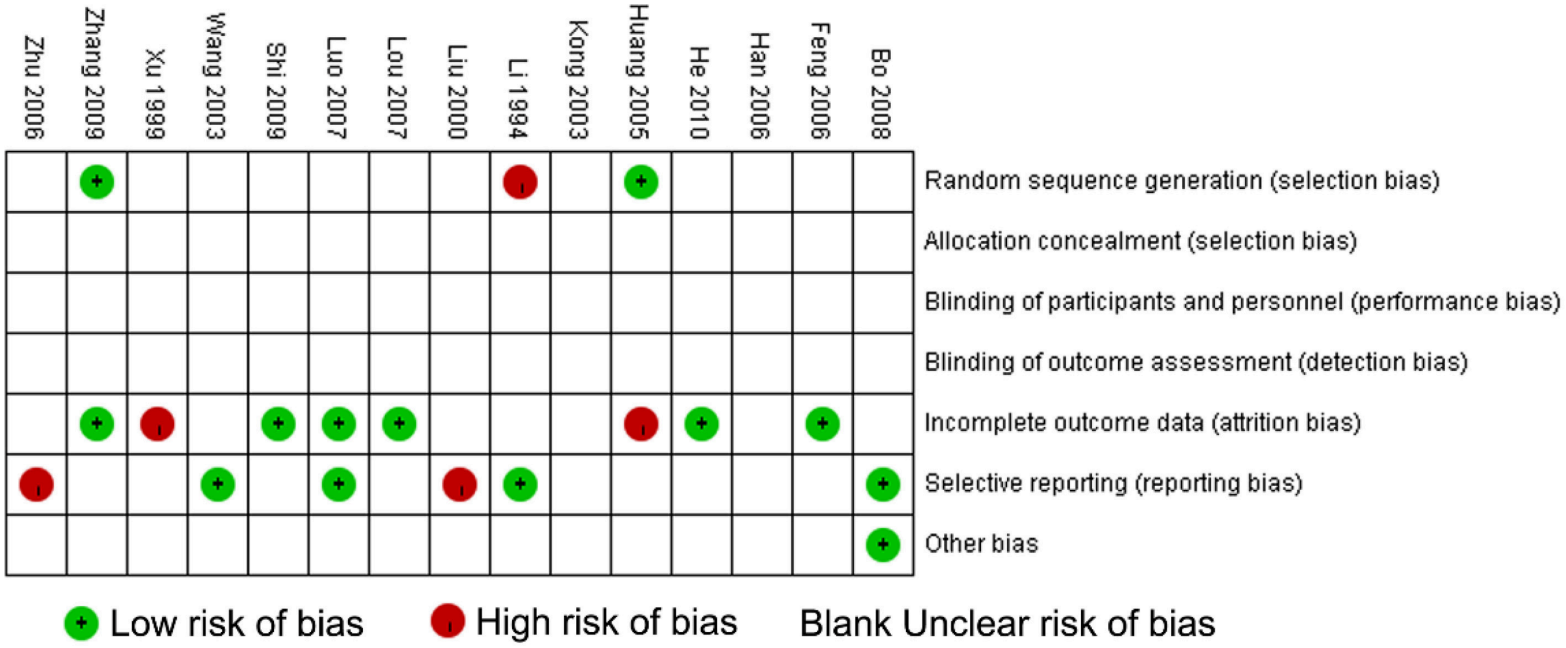
**Methodological quality assessment for risk of bias for each included study**. Risk of bias was used to assess the quality of RCTs, and the majority of studies were found to be of low quality.

The methodological quality of the 15 included trials was generally low. According to the Cochrane risk of bias estimation, a randomized allocation of participants was mentioned in all trials. However, only 2 trials (Huang and Yang, 2005; Zhang, 2009) mentioned an appropriate randomized method. Allocation concealment, blinding of participants and blinding of outcome assessment were not recorded in the 15 studies. Six of the 15 articles (Feng et al., 2006; Lou et al., 2007; Luo, 2007; Shi et al., 2009; Zhang, 2009; He, 2010) were at low risk of incomplete outcome data or essential data missing. Selective reporting was generally unclear. Only four articles (Li and Gu, 1994; Wang et al., 2003; Luo, 2007; Bo, 2008) reported research protocols, and the result of the detailed indices indicated a low risk of selective reporting. (Figure 3).

### Outcome measures with subgroup analysis

#### Efficacy rate of high-dose PRR-relevant formulae combined with essential treatment or single application vs. essential treatment

**Figure 4:**
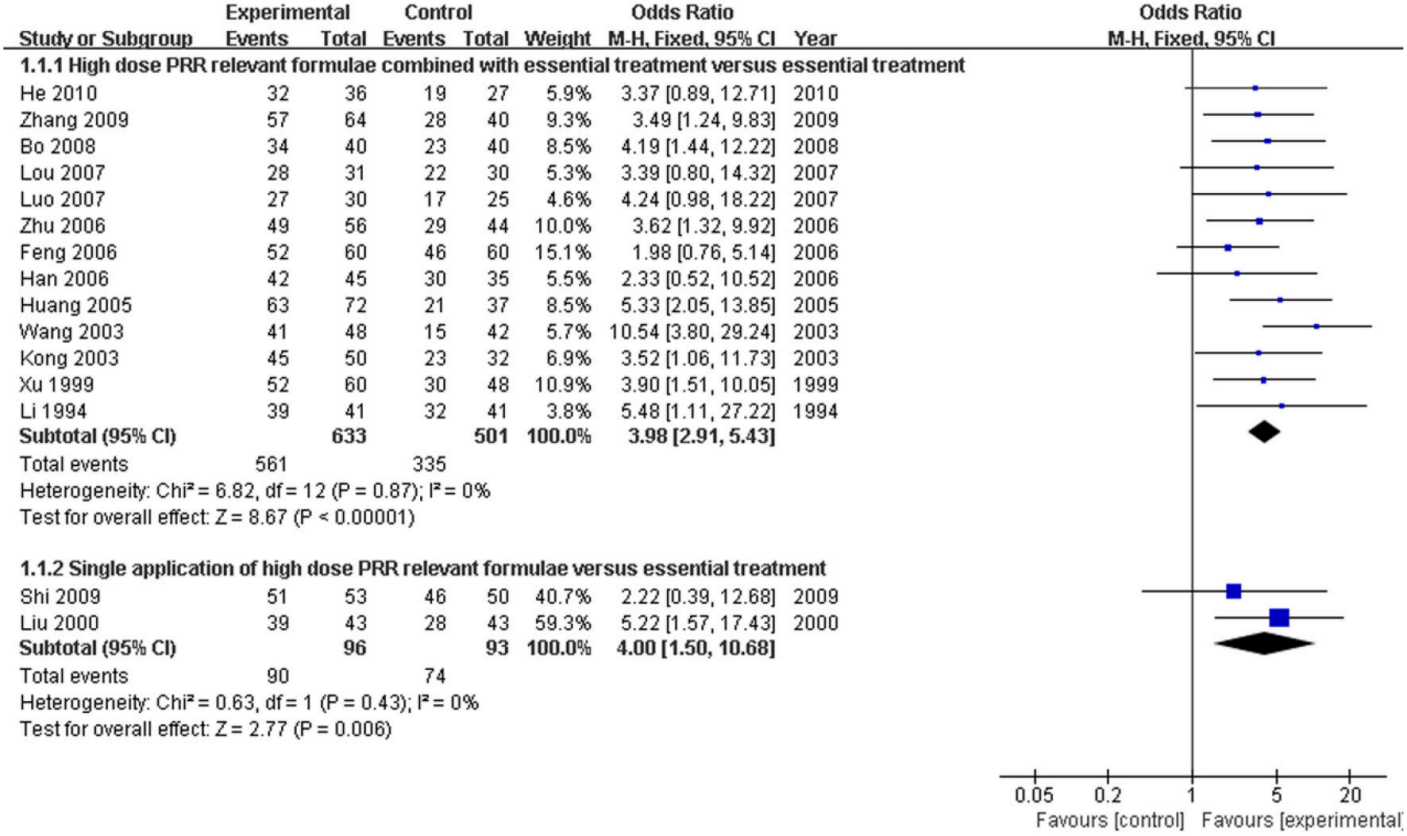
**Forest plot of high-dose PRR-relevant formulae with essential treatment or single application vs. essential treatment in viral hepatitis patients with hyperbilirubinemia**. (1) Study item displayed as first author with the publication year. (2) Subgroups were divided by formulae combined with essential treatment or single application. (3) I-squared and P are the criterion of the heterogeneity test, ♦ pooled odds ratio, —■— odds ratio, and 95% confidence interval.

Thirteen of the 15 articles described the application of formulae on high-dose PRR combined with essential treatment as the trial group. The remaining 2 RCTs (Liu, 2000; Shi et al., 2009) reported a single application of high-dose PRR formulae in the trial group. No heterogeneity was found in both analyses and a fixed effects model was used (*P* > 0.10). The results displayed as OR with 95% CI in the combination treatment and single application were [OR: 3.98, 95% CI (2.91, 5.43)] and [OR: 4.00, 95% CI (1.50, 10.68)], respectively. This revealed that formulae of high-dose PRR might promote the treatment efficacy for hyperbilirubinemia (*P* < 0.01; Figure 4).

#### Hyperbilirubinemia indices of high-dose PRR-relevant formulae vs. essential treatment

**Figure 5:**
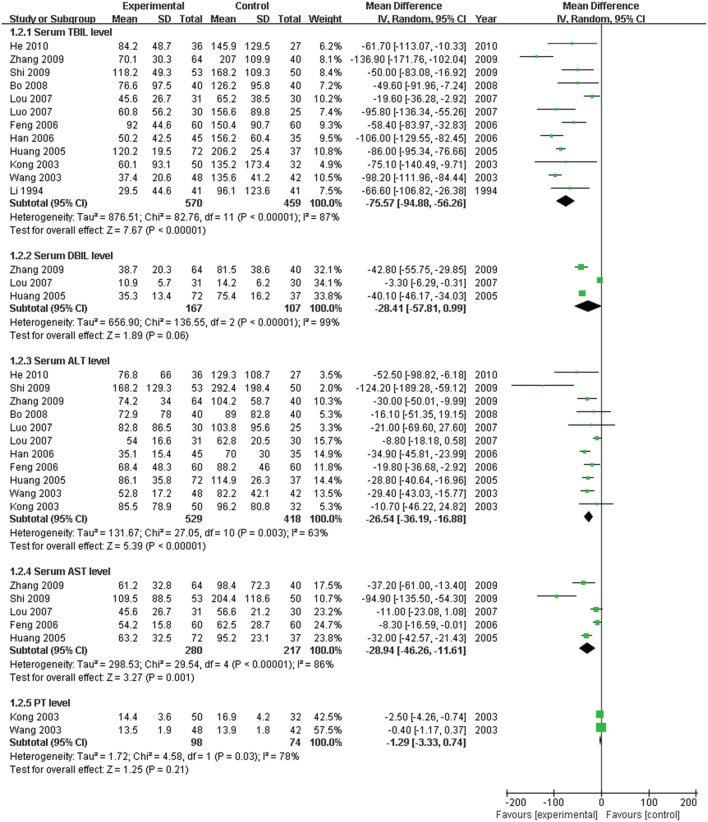
**Forest plot of TBIL, DBIL, ALT, AST, and PT in treatment**. (1) Study item displayed as first author with the publication year. (2) Subgroups were divided by indices of TBIL, DBIL, ALT, AST, and PT. (3) I-squared and P are the criterion of the heterogeneity test, ♦ pooled mean difference, —■— mean difference, and 95% confidence interval.

**Figure 6:**
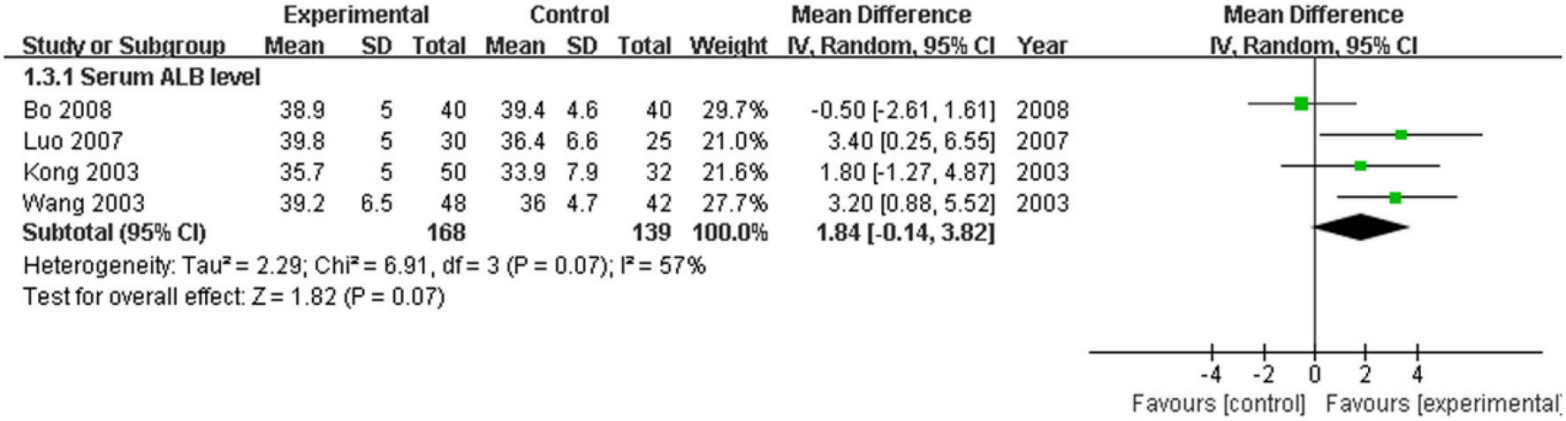
**Forest plot of ALB in treatment**. (1) Study item displayed as the first author with the publication year. (2) The index of serum ALB was assessed in RCTs. (3) I-squared and P are the criterion of the heterogeneity test, ♦ pooled mean difference, —■— mean difference, and 95% confidence interval.

**Figure 7:**
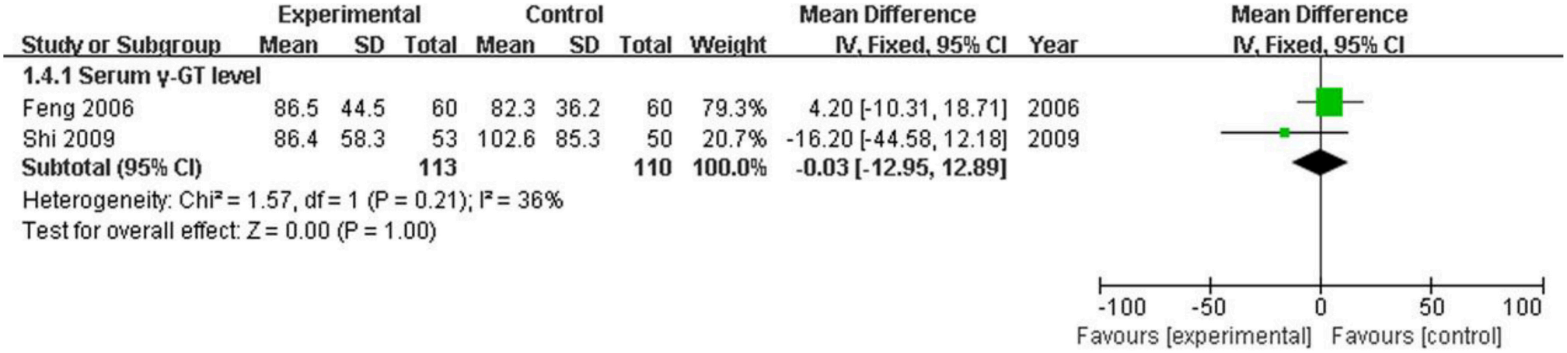
**Forest plot of γ-GT in treatment**. (1) Study item displayed as the first author with the publication year. (2) The index of serum γ-GT was assessed in RCTs. (3) I-squared and P are the criterion of the heterogeneity test, ♦ pooled mean difference, —■— mean difference, and 95% confidence interval.

The serum TBIL level, which is the direct and crucial index of hyperbilirubinemia, was described in 12 trials. In addition, 3 studies (Huang and Yang, 2005; Lou et al., 2007; Zhang, 2009) reported the serum DBIL level. The serum ALT level was measured in 11 articles, and five studies (Huang and Yang, 2005; Feng et al., 2006; Lou et al., 2007; Shi et al., 2009; Zhang, 2009) recorded the AST level. Serum ALB and γ-GT were reported in 4 (Kong et al., 2003; Wang et al., 2003; Luo, 2007; Bo, 2008) and two trials (Feng et al., 2006; Shi et al., 2009), respectively. Two trials (Kong et al., 2003; Wang et al., 2003) recorded the PT level. There was heterogeneity in the indices of TBIL, DBIL, ALT, AST, PT, and ALB (*P* < 0.10). Therefore, a random effects model was used for analysis. There was no heterogeneity (*P* > 0.10) in the index of γ-GT; the fixed-effect model was thus used. The MD with 95% CI of serum TBIL, ALT, and AST levels were [MD: −75.57, 95% CI (−94.88, −56.26)], [MD: −26.54, 95% CI (−36.19, −16.88)], and [MD: −28.94, 95% CI (−46.26,−11.61)], respectively, indicating a significant decrease in the pathologic index in the trial group compared with the control group (*P* < 0.01). The indices of serum DBIL, γ-GT, and PT were [MD: −28.41, 95% CI (−57.81, 0.99)], [MD: −0.03, 95% CI (−12.95, 12.89)], and [MD: −1.29, 95% CI (−3.33, 0.74)], respectively. There were no significant decreases in the trial group compared with the control group (*P* > 0.05). There was no difference between the trial group and control group for the serum index [MD: ALB 1.84, 95% CI (−0.14, 3.82)] (*P* > 0.05; Figures 5–7).

#### Efficacy rate of high-dose PRR-relevant formulae combined with essential treatment vs. essential treatment in hyperbilirubinemia patients with hepatitis B

**Figure 8:**
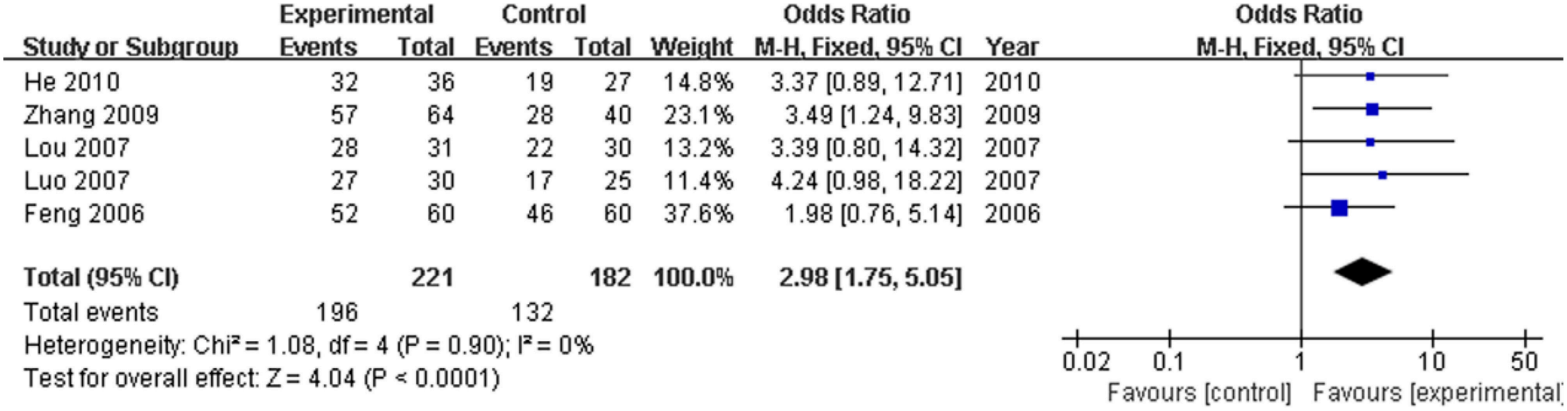
**Forest plot of high dose PRR relevant formulae with essential treatment vs. essential treatment in hyperbilirubinemia patients with hepatitis B**. (1) Study item displayed as the first author with the publication year. (2) Specific analysis was performed for hepatitis B virus patients with hyperbilirubinemia. (3) I-squared and P are the criterion of the heterogeneity test, ♦ pooled odds ratio, —■— odds ratio, and 95% confidence interval.

Hepatitis B virus is the most common and important cause of hyperbilirubinemia. In this study, the efficacy rate of formulae of high-dose PRR combined with essential treatment was specifically analyzed. There were five articles (Feng et al., 2006; Lou et al., 2007; Luo, 2007; Zhang, 2009; He, 2010) that reported hyperbilirubinemia patients with hepatitis B virus. Based on the fixed effects model (*P* > 0.10), the OR with 95% CI was [OR: 2.98, 95% CI (1.75, 5.05)]. The efficacy rate showed a significant increase in the trial group compared with the control group (Figure 8).

#### Efficacy rate of different ranges of high-dose PRR-relevant formulae combined with essential treatment vs. essential treatment in hyperbilirubinemia

**Figure 9:**
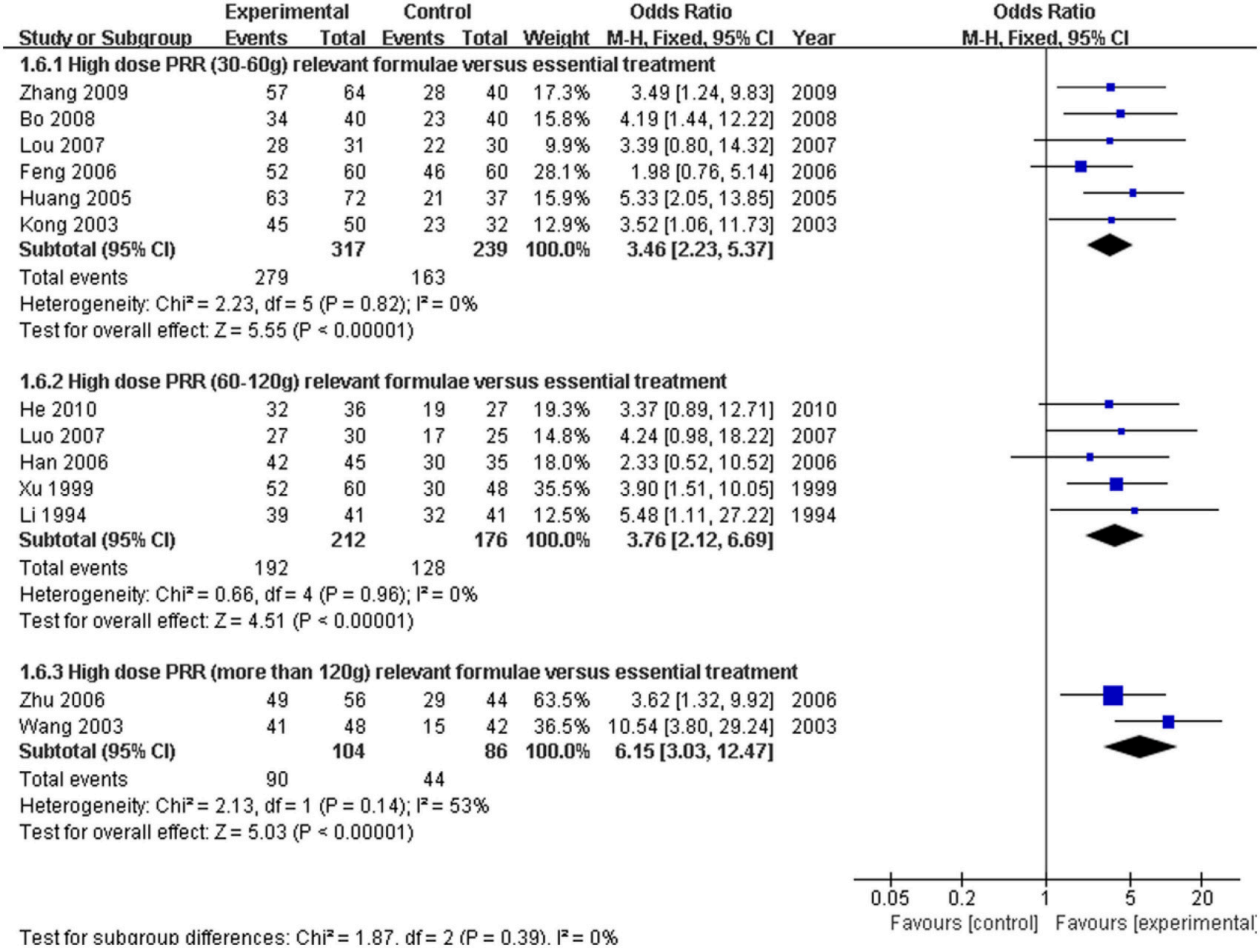
**Forest plot of the different ranges of high-dose PRR-relevant formulae combined with essential treatment vs. essential treatment alone**. (1) Study item displayed as the first author with the publication year. (2) Subgroups were classified as PRR 30–60 g, PRR 60–120 g and PRR greater than 120 g. (3) I-squared and P are the criterion of the heterogeneity test, ♦ pooled odds ratio, —■— odds ratio, and 95% confidence interval.

Because there was a wide range of high-dose PRR from 30 to 200 g, a subgroup analysis of different ranges of formulae of high-dose PRR was needed. We classified these studies into three subgroups (PRR in 30–60 g, PRR in 60–120 g, and PRR greater than 120 g) according to the dose application. There were six studies (Kong et al., 2003; Huang and Yang, 2005; Feng et al., 2006; Lou et al., 2007; Bo, 2008; Zhang, 2009) ascribed to the 30–60 g subgroup and five studies (Li and Gu, 1994; Xu et al., 1999; Han et al., 2006; Luo, 2007; He, 2010) ascribed to the 60–120 g subgroup. In addition, the remaining 2 studies (Wang et al., 2003; Zhu et al., 2006) were classified to the PRR greater than 120 g subgroup. On the basis of the fixed effect model (*P* > 0.10), the ORs with 95% CI of the three subgroups were [OR: 3.46, 95% CI (2.23, 5.37)] (PRR in 30–60 g), [OR: 3.76, 95% CI (2.12, 6.69)] (PRR in 60–120 g), and [OR: 6.15, 95% CI (3.03, 12.47)] (PRR more than 120 g), respectively. All three of the subgroups showed a significant increase in the efficacy rate of the trial group compared with the control group (*P* < 0.01). Furthermore, the efficacy was enhanced with an increasing dosage of PRR in relevant formulae (Figure 9).

### Adverse events

Among the 15 RCTs, two articles (Li and Gu, 1994; Bo, 2008) reported no side effects in clinical trials. Another three studies (Xu et al., 1999; Wang et al., 2003; Lou et al., 2007) observed side effects as light digestive tract symptoms in patients treated with formulae of high-dose PRR. However, the side effect did not influence the patients' efficacy or daily life. The remaining articles did not report whether there was a side effect. Despite the results suggesting that high-dose PRR is safe, more assessments on the safety of high-dose PRR are needed (Table 2).

### Publication bias

**Figure 10:**
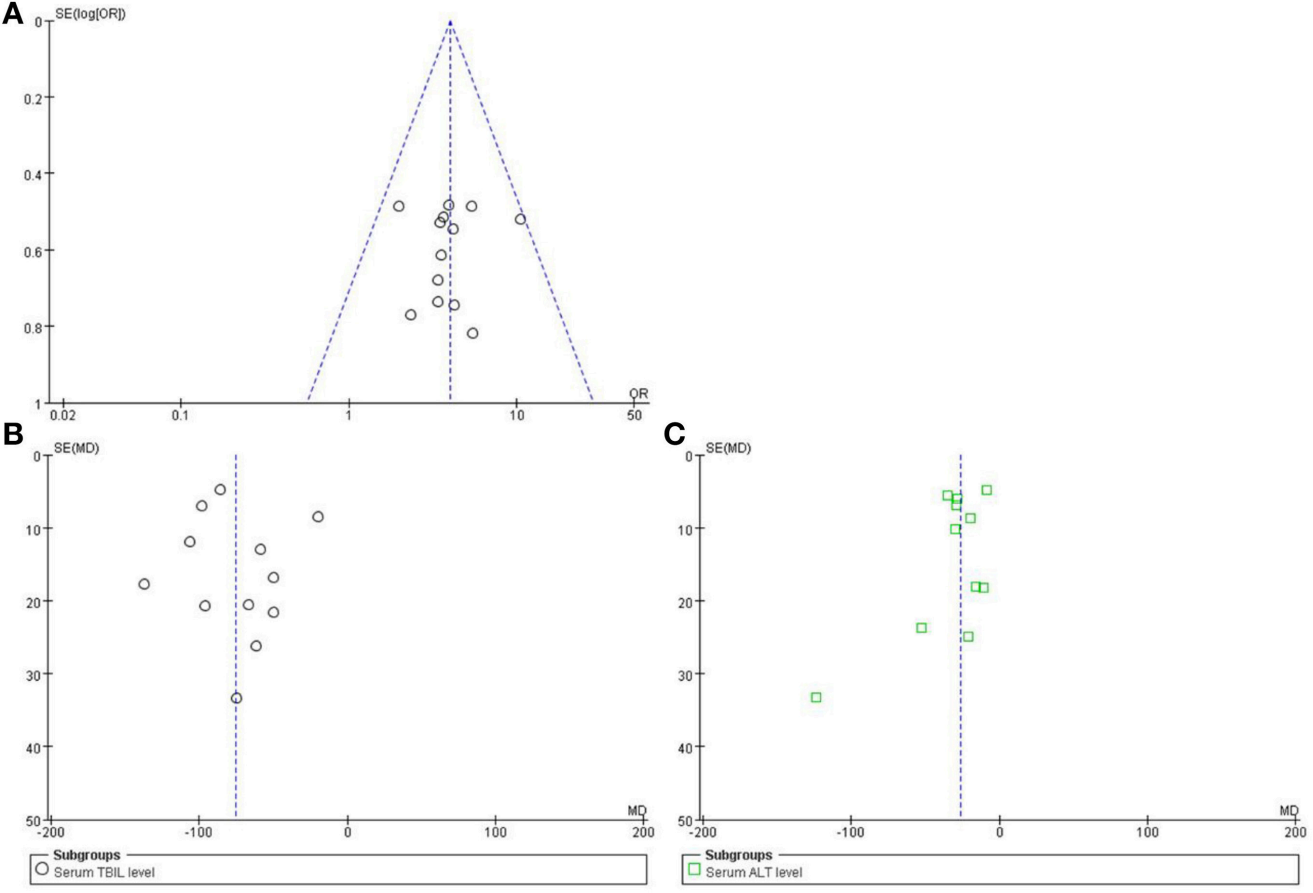
**Funnel plot of high-dose PRR-relevant formulae. (A)** The plot of efficacy rate, **(B)** serum index of TBIL, and **(C)** serum index of ALT was symmetric, suggesting that the publication bias was small.

A funnel plot was used to express the publication bias. The publication bias was explored when the indices or efficacy rate were above 9 cases. A funnel plot of the formulae of high-dose PRR combined with essential treatment vs. essential treatment was applied. Further, the indices of TBIL and ALT were also analyzed. The plots were symmetric, suggesting that there was no obvious publication bias (Figure 10).

## Discussion

As we mentioned, cholestatic hepatitis, and hyperbilirubinemia are both related to the dysfunction of bilirubin metabolism. However, there are several differences between cholestatic hepatitis. Cholestatic hepatitis is more concerned about the whole progression of the disease which could result in partial or complete blocking bile flow, high serum concentrations of bile acid, bilirubin, and liver injury. On the other side, hyperbilirubinemia could be widely expressed during the specific progression of various liver diseases and is more focused on the manifestation of high bilirubin. Moreover, the degree of bilirubin in hyperbilirubinemia is usually far more severe than in cholestatic hepatitis. In our former research, we have investigated the effect of high dose PRR relevant formulae on cholestatic hepatitis and found that this therapy could promote the efficacy (Ma et al., 2014). Therefore, according to the former result, we performed this meta-analysis on hyperbilirubinemia triggered by viral hepatitis. This systematic review included 15 RCTs; a total of 1323 participants with hyperbilirubinemia were screened from 4300 publications. All of the studies were based on high-dose PRR (30–200 g) serving as the primary component of the formulae. The treatment duration described by the RCTs ranged from 2 weeks to 60 days, and five of the 15 studies reported side effects. According to the outcome, a single application or a combination of high-dose PRR formulae promoted efficacious hyperbilirubinemia treatment. The indices of hyperbilirubinemia also verified the result. TBIL is considered a direct index of hyperbilirubinemia. The serum TBIL level in 12 trials was analyzed by the random effects model. The MD with the 95% CI of the serum TBIL level was [MD: −75.57, 95% CI (−94.88, −56.26)] and indicated that formulae of high-dose PRR significantly decreased the serum TBIL level compared with essential treatment. Furthermore, the serum ALT and AST levels, which are sensitive indices of hepatic function, also decreased. However, there were no differences in DBIL, γ-GT, PT, and ALB between the groups. This suggested that formulae of high-dose PRR might promote the regulation of the main indices of hyperbilirubinemia. Hepatitis B virus is the most common and important cause of hyperbilirubinemia. Therefore, the efficacy of high-dose PRR formulae on hyperbilirubinemia triggered by hepatitis B was specifically analyzed in this study. The result showed a significant increase in the efficacy rate in the trial group compared with the control group. This suggested that formulae of high-dose PRR might promote efficacious treatment of the hyperbilirubinemia caused by hepatitis B. Because there was a wide range of high-dose PRR, from 30 to 200 g, we performed an analysis on the efficacy rate of different ranges of high-dose PRR formulae. All three subgroups showed a significant increase in the efficacy rate of the trial group. Furthermore, the efficacy enhanced as the dosage of PRR in the relevant formulae was increasing. There were a few patients with light side effects of the digestive tract in the trial group. However, it did not influence the treatment progress and efficacy. A funnel plot was applied to assess the publication bias. The result indicated that little publication bias occurred in terms of efficacy and indices. This systematic review illustrates that formulae of high-dose PRR are safe to promote the efficacious treatment of hyperbilirubinemia.

Hyperbilirubinemia, presenting with severe jaundice, is one of the most common manifestations in viral hepatitis patients. It usually leads to severe necrosis of hepatocytes over a chronic period (Wang, 2010). It is well-known that a variety of definite factors, including infection, drug abuse, autoimmunity, and heredity, are able to cause hyperbilirubinemia. As for the mechanism, researchers have mainly focused on disorders of bilirubin metabolism such as uptake, synthesis, and excretion (Nie, 2006). In detail, repeated inflammation in viral hepatitis always acts as the trigger of jaundice and ultimately results in hepatocyte damage and even necrosis. Once hepatocytes are damaged, the metabolism of bilirubin is disturbed, thus causing bilirubin accumulation. If the high concentration of bilirubin fails to be eliminated, it accumulates such that hyperbilirubinemia or jaundice occurs. Moreover, a micro-imbalance would also occur in hepatocytes, such as inhibition of Na^+^-K^+^-ATPase, down regulation of NTCP and OATP expression, and deregulation of CAR and PXR expression. All of these consequences lead to bilirubin deposition in hepatocytes and jaundice and can cause worsening of hepatocyte damage (Paumgartner and Beuers, 2002; Wagner et al., 2011; Wlcek et al., 2013). According to the mechanism, hyperbilirubinemia is associated with various tissues and targets. Current western medical treatment for hyperbilirubinemia mainly focuses on the combination of alleviating jaundice, liver protection, lowering transaminase levels, nutrition support, etc. However, it lacks comprehensive adjustment and has unstable efficacy. In contrast, complementary and alternative medicine such as TCM for the treatment of hyperbilirubinemia often obtains positive results.

*PRR* has been widely used in TCM practice for thousands of years for the treatment of blood stasis by targeting the liver. Current studies indicate that PRR and its active components consistently exhibit wide pharmacological effects such as vasodilatation of the thoracic aorta (Jin et al., 2010), liver protection (Zhao et al., 2013), an anti-allergic effect (Lee et al., 2008), anti-inflammation, and immunoregulation (He and Dai, 2011). According to the TCM theory and modern pharmacology, the original clinical practice of formulae of high-dose PRR to treat hyperbilirubinemia has been gradually applied in recent years (Zhu, 2011). In addition, some studies may partially reveal the mechanism of this application. PRR is able to promote bile acid excretion, enhance the activity of UDPGT and decrease the TXB_2_ level (Wang, 1986; Lei et al., 1988). In this aspect, PRR is effective in decreasing bilirubin. Although, there is efficacy on hyperbilirubinemia, it remains unclear why PRR should be used at such a high dose. Clinical studies have shown that there is a significant difference in efficacy between the high-dose treatment group (30 g) and the normal-dose treatment group (10 g). The efficacy of the high-dose treatment group is markedly higher than the normal-dose treatment group in alleviating hyperbilirubinemia (*P* < 0.01) (Luo, 2007). This indicates that low-dose PRR cannot effectively treat hyperbilirubinemia compared with a high dose. This is likely related to the characteristic active compounds in PRR. Our previous study demonstrated that the bioavailability of paeoniflorin and albiflorin in PRR is extremely low (Jiang et al., 2012). Herein, a high dose of PRR shows stable treatment efficacy for the alleviation of hyperbilirubinemia, which was not found at low doses. According to these results, formulae of high-dose PRR might help uncover the crucial and specific function of this therapy for hyperbilirubinemia.

Currently, increasing attention has focused on TCM. Due to the holistic concept and treatment based on syndrome differentiation, TCM emphasizes formulae employment instead of a single drug application in treating diseases. Many studies demonstrate that formulae with favorable efficacy for specific diseases regulate body function in various organs and tissues in a network manner (Li et al., 2007; Li and Zhang, 2013). Therefore, further studies to illustrate the potential mechanism should be explored.

## Conclusion

Formulae of high-dose PRR might safely promote the efficacious treatment of hyperbilirubinemia and are thus a promising treatment approach. For acceptance by widespread practitioners, more rigorously designed multicenter, double-blind, randomized, and large-scale controlled trials are required for further investigation.

## Author contributions

YH, XM, and JW analyzed the data and wrote the manuscript. YZ, ZC, and WZ collected and prepared samples. JW, JW, LS performed the analyses. YZ and XX designed the study and amended the paper.

### Conflict of interest statement

The authors declare that the research was conducted in the absence of any commercial or financial relationships that could be construed as a potential conflict of interest.

## Abbreviations

PRR: *Paeoniae Radix Rubra*
ALT: alanine aminotransferase
AST: aspartate aminotransferase
TBIL: total bilirubin
UDCA: ursodeoxycholic acid
TCM: traditional Chinese medicine
RCTs: randomized control trials
VMIS: VIP medicine information system
CBM: Chinese Biomedical Database
CNKI: China National Knowledge Infrastructure
VHPTP: Viral Hepatitis Prevention and Treatment Programs
γ-GT: γ-glutamyl transferase
DBIL: direct bilirubin
PT: prothrombin time
MD: mean difference
OR: odds ratio.
